# Atypical cyclin P regulates cancer cell stemness through activation of the WNT pathway

**DOI:** 10.1007/s13402-021-00636-7

**Published:** 2021-10-04

**Authors:** Abril Sánchez-Botet, Eva Quandt, Núria Masip, Rubén Escribá, Laura Novellasdemunt, Laura Gasa, Vivian S. W. Li, Ángel Raya, Josep Clotet, Mariana P. C. Ribeiro

**Affiliations:** 1 Department of Basic Sciences, Faculty of Medicine and Health Sciences, Universitat Internacional de Catalunya, Josep Trueta, s/n, 08195 Sant Cugat del Vallès, Barcelona, Spain; 2 Regenerative Medicine Program, Bellvitge Institute for Biomedical Research (IDIBELL) and Program for Clinical Translation of Regenerative Medicine in Catalonia (P-CMRC), L’Hospitalet del Llobregat, Barcelona, Spain; 3 Centre for Networked Biomedical Research on Bioengineering, Biomaterials and Nanomedicine (CIBER-BBN), Madrid, Spain; 4 Stem Cell and Cancer Biology Laboratory, The Francis Crick Institute, London, UK; 5 Catalan Institution for Research and Advanced Studies (ICREA), Barcelona, Spain

**Keywords:** Cancer stem cells (CSCs), WNT, Colorectal cancer, Lung cancer, Breast cancer, Cyclin P

## Abstract

**Purpose:**

Cancer stem cells represent a cancer cell subpopulation that has been found to be associated with metastasis and chemoresistance. Therefore, it is vital to identify mechanisms regulating cancer stemness. Previously, we have shown that the atypical cyclin P (CCNP), also known as CNTD2, is upregulated in lung and colorectal cancers and is associated with a worse clinical prognosis. Given that other cyclins have been implicated in pluripotency regulation, we hypothesized that CCNP may also play a role in cancer stemness.

**Methods:**

Cell line-derived spheroids, ex vivo intestinal organoid cultures and induced-pluripotent stem cells (iPSCs) were used to investigate the role of CCNP in stemness. The effects of CCNP on cancer cell stemness and the expression of pluripotency markers and ATP-binding cassette (ABC) transporters were evaluated using Western blotting and RT-qPCR assays. Cell viability was assessed using a MTT assay. The effects of CCNP on WNT targets were monitored by RNA-seq analysis. Data from publicly available web-based resources were also analyzed.

**Results:**

We found that CCNP increases spheroid formation in breast, lung and colorectal cancers, and upregulates the expression of stemness (CD44, CD133) and pluripotency (SOX2, OCT4, NANOG) markers. In addition, we found that CCNP promotes resistance to anticancer drugs and induces the expression of multidrug resistance ABC transporters. Our RNA-seq data indicate that CCNP activates the WNT pathway, and that inhibition of this pathway abrogates the increase in spheroid formation promoted by CCNP. Finally, we found that CCNP knockout decreases OCT4 expression in iPSCs, further supporting the notion that CCNP is involved in stemness regulation.

**Conclusion:**

Our results reveal CCNP as a novel player in stemness and as a potential therapeutic target in cancer.

**Supplementary Information:**

The online version contains supplementary material available at 10.1007/s13402-021-00636-7.

## Introduction

Cancer is a major public health issue with lung, breast and colorectal cancers representing the most common ones worldwide [1, 2]. Together with prostate cancer, they represent half of the overall burden of cancer in Europe [2] and, although treatment protocols have improved over the years, most patients presenting late-stage cancer will relapse.

A major cause underlying cancer relapse is the failure of current treatments to eradicate cancer stem cell (CSC) populations. The CSC paradigm postulates that tumors, as normal tissues, are organized hierarchically, in which CSCs are defined by unlimited proliferation and self-renewal capabilities [3, 4]. CSCs have recently been considered as architects of the tumor environment supporting tumor heterogeneity, thereby facilitating tumor fitness, therapeutic resistance and metastasis [5–8]. Therefore, the identification of regulators of cancer stemness will be vital for the development of effective therapeutic strategies.

Increasing evidence indicates that cell cycle dynamics are implicated in cell differentiation and stemness through the regulation of gene networks at different levels [9]. Thus, it is not surprising that the master regulators of cell cycle progression, the cyclin-dependent kinases (CDKs) and their regulatory subunits, the cyclins, play critical roles in pluripotent state regulation. It has been shown that embryonic stem cells display high levels of CDK2 activity, particularly at the onset of differentiation [10–12]. Moreover, it has been reported that CDK2 inhibition decreases the levels of OCT4 [13] and leads to loss of the pluripotent state [14, 15]. More recently, it has been shown that cyclins D and E directly regulate the pluripotent state through the phosphorylation of NANOG, OCT4 and SOX2 [16].

Interestingly, the classification of the cyclin family of proteins has recently been revised to include a subfamily of atypical cyclins, which are characterized by structural and interactor pattern particularities [17]. Although the function of atypical cyclins is still poorly characterized, the expression of members of this subfamily is often deregulated in cancer and may have prognostic significance [18]. Indeed, atypical cyclins seem to participate in the regulation of cell proliferation, cell migration, treatment response, epithelial-to-mesenchymal transition (EMT) and stemness [18]. Regarding the latter, cyclins G1, G2 [19, 20], Y and Y-like1 [21] have garnered most attention, but it is conceivable that other atypical cyclins are implicated in pluripotency regulation as well. Remarkably, the atypical cyclin P (CCNP), also known as CNTD2, has been found to promote EMT in colorectal cancer and to endow cells with anchorage-independent growth [22]. Given the known correlation between EMT and CSC plasticity, we hypothesized that CCNP may be implicated in cancer stemness.

Here, we report that CCNP promotes sphere formation, expression of stemness and pluripotency markers and drug resistance in cancer cells. Mechanistically, we found that the effects of CCNP on stemness regulation are mediated through activation of the WNT pathway.

## Material and methods

### Cell lines and reagents

All cells were grown in Dulbecco's Modified Eagle Medium (DMEM), with the exception of NCI-H1395 cells that were grown in Roswell Park Memorial Institute (RPMI-1640) medium (Sigma-Aldrich). The media were supplemented with 10% heat-inactivated fetal bovine serum (FBS, Sigma-Aldrich), 1% glutamax (Biowest, Nuaillé) and 1% penicillin/streptomycin (Sigma-Aldrich). Cells were grown in a humidified air cabin at 37 °C and 5% CO_2_. A full list of cell lines used with catalog numbers is provided in Supplementary Table S1.

### Human intestinal organoids

Organoids were kindly provided by Vivian Li’s lab. Crypts were isolated and plated in drops of basement membrane extract (BME) as previously described [23]. After BME polymerization, culture medium was added. The culture medium was prepared as described elsewhere [23], except that CHIR (R&D Systems) and RhoKinase inhibitor (Sigma) were also added.

For counting purposes, human organoids were single cell dissociated using TrypLE Express and mechanical dissociation with fire-polished Pasteur pipettes, after which they were counted using an Automated Cell Counter (Thermo Fisher).

### Sphere formation assays

Single cells were seeded in Matrigel (Corning) at a density of 300 cells per well or in ultra-low attachment plates (Corning) at a density of 5000 cells per well and incubated for 10 or 7 days, respectively, in serum-free medium (DMEM/F12) supplemented with 2% B27 (Gibco, Thermo Fisher Scientific), 20 ng/ml epidermal growth factor (EGF) (PeproTech) and 20 ng/ml fibroblast growth factor (FGF) (PeproTech). Spheres grown in Matrigel were counted after 10 days of culture under a 20 × objective; only spheres with a diameter > 100 µm were considered.

### Viral cloning, transduction and infection

Lentiviral vectors were prepared as previously described [24]. Viral titers were determined by transducing HEK293-T cells with serial virus dilutions, after which optimal multiplicities of infection (MOI) were determined for each cell line: A549 (MOI = 15), LoVo (MOI = 20) and MCF7 (MOI = 30). For overexpression studies, cells were seeded and transduced 24 h later. Medium was added the following day and, 4 days later, cells were analyzed or seeded for sphere generation.

For overexpression in human organoids, pre-warmed 24-well plates were coated with BME and incubated at 37 °C. Human organoids were dissociated, after which 50,000 cells were mixed with 300 μl organoid medium, virus (MOI = 50) and polybrene (5 μg/ml, Sigma), and then added to prepared plates. After 24 h, media were removed and 80 μl fresh BME was added. After incubation at 37 °C, 500 μl organoid medium was added.

### Quantitative reverse transcription PCR (RT-qPCR) analysis

Total RNA was extracted, after which RT-qPCR was performed as described elsewhere [22]. The primers used are listed in Supplementary Table S2. The obtained values were normalized using 18S as a housekeeping gene and calculated according to the 2^−ΔΔCt^ method.

### Western blot analysis

Total cell extract preparation and immunodetection were carried out as previously described [24], except that 30 µg of total protein was separated by 4–15% SDS-PAGE (BioRad). The primary antibodies used are listed in Supplementary Table S3. Protein bands were analyzed using the LI-COR Image Studio Software program and normalized using Ponceau staining.

### Small interfering RNA (siRNA) transfection

Cells were seeded in 24-well plates at a density of 8 × 10^4^ cells per well. The following day, transfections were carried out using Lipofectamine 2000 (Thermo Fisher Scientific) and 50 nM CCNP siRNA (SR312715A and SR312715B, OriGene) or a scrambled negative control (OriGene). After 72 h of incubation, CCNP knockdown was monitored by RT-qPCR, and cells were analyzed or seeded for sphere generation.

For human organoid experiments, they were dissociated and 100,000 cells were mixed with 100 μl BME and plated in a pre-warmed 24-well plate, which was then incubated at 37 °C. Next, 50 nM CCNP siRNA or negative control were mixed with Lipofectamine 2000 in 500 μl Optimum medium and added to the culture medium. Transfection efficiencies were monitored in parallel using a fluorescent siRNA (SR3002, Origene).

### Immunofluorescence assay

LoVo cells transduced with an empty vector or a CCNP expression construct were seeded on poly-lysine coated coverslips in 24-well plates at a density of 25,000 cells/well and, 48 h later, the cells were fixed for 15 min in cold 4% paraformaldehyde at 4 °C. Next, the cells were blocked for 1 h with PBS containing 2.5% Goat Serum and 0.1% Triton, followed by 1 h incubation with anti-CD44 (189,524, Abcam) or anti-CD133 (64326, Cell signaling) antibodies at dilutions of 1:250 or 1:500, respectively. Coverslips were then washed several times with PBS and incubated in the dark with an anti-rabbit fluorescent secondary antibody (Invitrogen, A11011) at a dilution of 1:1000.

### RNA-seq assay

LoVo cells were transduced with a CCNP-overexpression construct or an empty control or transfected with siRNA targeting CCNP or a scrambled negative control as described above. Next, extracted mRNA was analyzed by Sequentia (https://www.sequentiabiotech.com/) using the online platform AIR (www.transcriptomics.cloud) as described elsewhere [25].

### Culture of human iPSCs

The use of human iPSCs in the present work was approved by the competent authorities (Commission on Guarantees concerning the Donation and Use of Human Tissues and Cells of the Carlos III Spanish National Institute of Health). A control human iPSC line generated from a healthy individual (codename FiPS Ctrl1-mR5F-6, registered in the National Stem Cell Bank, Carlos III Spanish National Institute of Health) was used for the generation of human iPSC-CCNP knock-outs (KOs). Cells were cultured in 1:100 Matrigel (354277, Corning)-coated dishes and routinely maintained in mTeSR1 medium (Stem Cell Technologies). The medium was changed every day. Cells were split by dissociation with 0.5 mM ethylenediaminetetraacetic acid (EDTA) (AM9260G, Invitrogen) for 2 min at 37 °C and seeded on Matrigel-coated plates. Cell lines were used at passages 20–45.

### CRISPR/Cas9 genome editing of human iPSCs

To generate a large deletion in the CCNP gene, a dual guide RNA(sgRNA)/Cas9 strategy was used. Candidate sgRNAs were selected in the region 250 bp either upstream or downstream of exon 2 and at the end of the 3’UTR using Benchling (https://www.benchling.com/crispr/). The following sgRNAs were selected: 5’-TCTAGGTGAGGACACGTAAG-3' and 5’-GGACCGGAATTCGACCAATG-3’. For CRISPR/Cas9 ribonucleoprotein (RNP) complex assembly, 300 pmol dual annealed cr:tracrRNA (Integrated DNA Technologies) was mixed with 40 pmol Cas9 nuclease (Thermo Fisher) and incubated at room temperature for 10 min. The assembled RNP complexes were mixed with 250,000 dissociated cells in 20 μl P3 primary Cell solution (Lonza). Next, the cells were placed into Nucleocuvette strips (Lonza) and transfected using an Amaxa 4D-Nucleofector system (Lonza). The transfected cells were then seeded onto 48-well plates containing pre-warmed mTeSR1 medium supplemented with 10 μM Rho kinase inhibitor (RI) (Y-27632, STEMCELL Technologies). After 3–4 days, cells were harvested, and approximately 15,000 cells were seeded in Matrigel-coated 10-cm plates containing RI-supplemented mTesR1 medium and maintained for 10–14 days. Next, individual colonies were manually picked and expanded. Genomic DNA was extracted using a DNeasy Blood and Tissue Kit (Qiagen) following the manufacturer’s instructions after which PCR-based genotyping was performed. The following primer sequences were used (Fwd: 5ʹ-TCTGGGAACCTAGAGGGAGA-3ʹ; Rev: 5 ʹ-GGCAACAAGAACGAAACTCC-3ʹ). Sanger sequencing was performed to confirm successful deletion, and colonies with the desired genotype were further expanded and cryopreserved.

### MTT viability assay

Cells previously transduced with an empty vector or a CCNP-expression construct, as described above, were seeded in 24-well plates and treated with drugs 24 h later. After 3 days, the MTT viability assay was performed as described elsewhere [26]. Drugs were purchased from Sigma, with the exceptions of gefitinib (Cell Signaling) and oxaliplatin (Cayman).

### Depmap analyses

Gene expression data were obtained from the Depmap portal (depmap.org/portal). Samples were ranked according target gene expression and divided into four even groups. Comparisons were made between samples with the lowest 25% expression and the highest 25% expression, as described elsewhere [27].

### Statistical analysis

Data are presented as the mean ± standard error of the mean (SEM) with *p*-values ***p < 0.001, **p < 0.01 or *p < 0.05 considered to indicate significant differences. Statistical significances were determined using the Mann–Whitney test for non-parametric data or the T-student test for the parametric ones. Statistical analyses were conducted using GraphPad Prism 5 and the Statistical Package for Social Sciences (SPSS) 21.

## Results

### CCNP promotes CSC-like properties

To select cancers in which CCNP plays a relevant role, we compared the mRNA levels between normal and tumor tissues in a large number of patients using the Gene Expression Profiling Interactive Analysis (GEPIA) database (http://gepia.cancer-pku.cn/) [28]. The mRNA levels of CCNP were found to be higher in tumor tissues than in normal ones, especially in colon, lung and breast cancers (Supplementary Fig. S1A). Noteworthy, we previously showed that high levels of CCNP are correlated with a worse clinical prognosis in lung cancer [24]. Moreover, we found that high CCNP levels correlate with a decreased overall survival in breast cancer patients (*p* = 0.002) (Supplementary Fig. S1B). Considering the high prevalence and the prognostic significance of CCNP in these cancer types, we decided to proceed with our studies in cellular models of lung, colon and breast cancer.

**Fig. 1:**
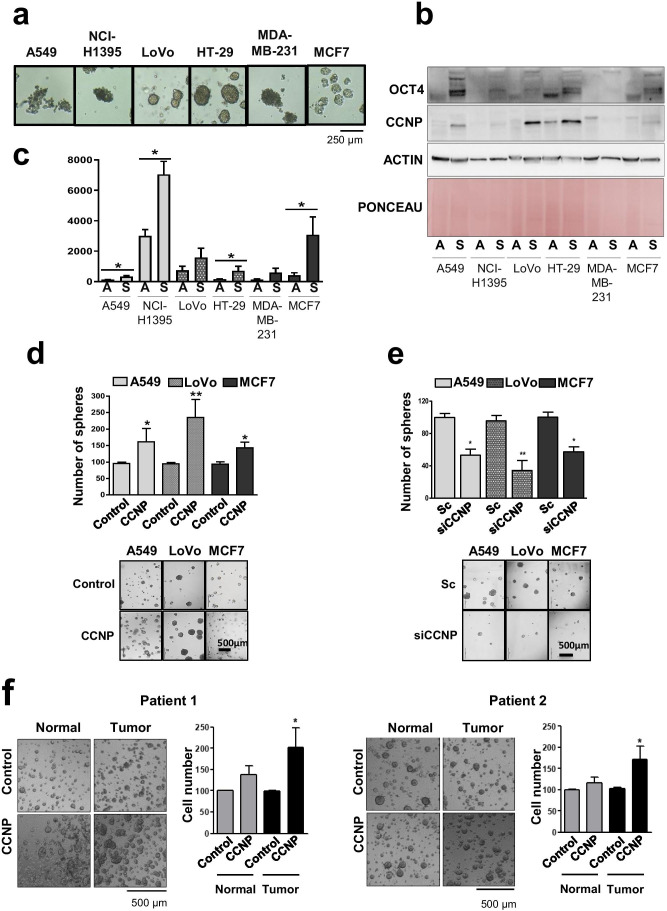
CCNP promotes CSC-like properties. **a** Spheroids were generated from lung (A549 and NCI-H1395), colon (LoVo and HT-29) and breast (MDA-MB-231 and MCF7) cancer cell models. **b** CCNP protein expression was analyzed by Western blotting in adherent (A) and spheroid (S) cultures. **c** RT-qPCR analysis of CCNP expression in adherent cells (A) and spheroids (S). The columns represent the mean ± SEM of five independent experiments. **d** Cell lines were transduced with empty lentivirus (control) or with a CCNP expression construct. Subsequently, cells were seeded in Matrigel. **e** Cells were transfected with 50 nM scrambled control (Sc) or siRNA targeting CCNP and seeded for spheroid formation 72 h later. In both **d** and **e**, the numbers of spheroids were determined ten days later, and the columns represent the mean ± SEM of five independent experiments performed in triplicate. The results are expressed as percentage of the control. **f** Patient normal- and tumor-derived organoids were transduced with empty lentivirus (control) or with a CCNP expression construct. Organoids were later dissociated into single cells and counted. *p < 0.05, **p < 0.01, Mann–Whitney test

To investigate whether CCNP is involved in cancer cell stemness, we cultured 6 cancer cell lines as spheroids to enrich for stemness characteristics (Fig. 1A and S2) and compared the protein expression levels of CCNP in the spheroids and parental cell lines (Fig. 1B). We found that CCNP was remarkably upregulated in spheroids compared to the corresponding adherent cells in some of the cellular models, especially in A549 lung cancer cells and HT-29 and LoVo colon cancer cells (Fig. 1B). We also monitored the mRNA expression levels of CCNP in the spheroids and parental cell lines by RT-qPCR (Fig. 1C). We selected 18S as a housekeeping gene given that its level is stable in both adherent cells and spheroids [29]. In line with the protein expression results, we found that the CCNP mRNA level was significantly increased in spheroids obtained from A549 (*p* = 0.0366), NCI-H1395 (*p* = 0.0286), HT-29 (*p* = 0.0159) and MCF7 (*p* = 0.0087) cells (Fig. 1C).

Next, we investigated whether CCNP upregulation increases the sphere numbers in selected cell lines of each tumor type. To this end, the cells were transduced with an empty (control) construct or CCNP expression constructs (Supplementary Fig. S3A), after which sphere numbers were determined one week later. CCNP overexpression in the three cell lines tested significantly increased the number of spheres in Matrigel (*p*-values: A549 = 0.0159; LoVo = 0.0079; MCF7 = 0.019) (Fig. 1D). Likewise, CCNP downregulation using siRNA (Supplementary Fig. S3B) significantly decreased the sphere numbers (*p*-values: A549 = 0.0223; LoVo = 0.0067; MCF7 = 0.0242) (Fig. 1E), supporting the notion that CCNP may play a critical role in cancer stemness.

Next, we investigated whether the results obtained with human cancer cell lines could be recapitulated in ex vivo intestinal organoid cultures, which were compared to genetically engineered mouse models or patient-derived xenografts [30]. Organoids obtained from 2 patients were transduced with an empty lentiviral vector (control) or CCNP expression constructs (Supplementary Figs. S3C-D). The resulting organoids were mechanically dissociated, after which cell numbers were determined. We found that CCNP overexpression increased the number of cells in tumor-derived organoids (*p*-values: patient 1 = 0.0265; patient 2 = 0.0294), but not in normal tissue-derived organoids (*p*-values: patient 1 = 0.0765; patient 2 = 0.6579) (Fig. 1F). Together, these results indicate that the increased expression of CCNP as observed in cancer patients [22, 24] may contribute to stemness-like phenotypes.

### CCNP regulates the expression of stemness markers and pluripotency factors in cancer cells

**Fig. 2:**
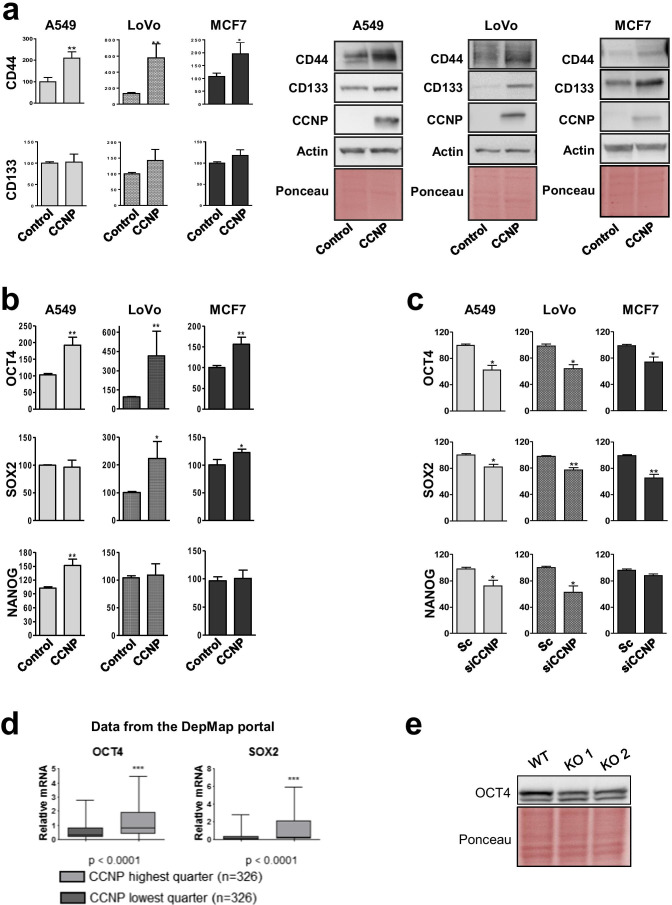
CCNP regulates the expression of stemness markers and pluripotency factors in cancer cells. **a** Protein expression of CD44 and CD133 was analyzed by Western blotting in adherent cell lines overexpressing CCNP and in control cells and standardized with Ponceau staining. The columns represent the mean ± SEM of ten independent experiments. Representative images of Western blot analysis performed after cell transduction with an empty lentiviral vector (control) or CCNP expression constructs are shown in the right panel. **b** Cells were transduced with empty lentivirus (control) or with a CCNP expression construct and, subsequently, seeded in Matrigel. After 10 days, the levels of pluripotency factors were evaluated by RT-qPCR. **c** Cells were transfected with siRNA directed against CCNP or scrambled control (Sc) and, after 72 h, the expression of pluripotency markers was evaluated by RT-qPCR. In both **b** and **c**, columns represent the mean ± SEM of five independent experiments performed in triplicate. *p < 0.05, **p < 0.01 versus control, Mann–Whitney test. The results are expressed as percentage of the control. **d** Data from the Depmap portal was used to assess the correlation between CCNP and OCT4 and SOX2 expression in human cell lines (n = 326 for each quarter). NANOG was excluded from this analysis as the expression level was too low to draw any reliable conclusions. Data accessed on June 10^th^, 2020. ***p < 0.001, Mann–Whitney test. **e** OCT4 protein expression was monitored in wild type and two iPSC clones lacking CCNP expression obtained by CRISPR-Cas9 mediated knockout (KO)

Since markers such as CD44 and CD133 have been used to facilitate normal and cancer stem cell identification [31], we next examined their protein expression in adherent cell lines following CCNP upregulation using lentiviral vectors. We found that CD44 expression was significantly increased in the three cell lines overexpressing CCNP (*p*-values: A549 = 0.010; LoVo = 0.0081; MCF7 = 0.0219) (Fig. 2A). The effect of CCNP upregulation on CD44 protein expression was confirmed by immunofluorescence in LoVo cells (Supplementary Fig. S4).

Next, we evaluated the effects of CCNP upregulation on mRNA expression of the pluripotency factors OCT4, NANOG and SOX2. Considering that the baseline levels of pluripotency factors in adherent cells is typically low, we conducted our experiments in spheres rather than in adherent cells lines to obtain more reliable measurements. We found that CCNP overexpression increased the levels of OCT4 in the three cell lines tested (*p*-values: A549 = 0.0043; LoVo = 0.0048; MCF7 = 0.0062), whereas the effects on NANOG (*p*-values: A549 = 0.0079; LoVo = 0.936; MCF7 = 0.7923) and SOX2 (*p*-values: A549 = 0.8861; LoVo = 0.0172; MCF7 = 0.0499) expression were cell line dependent (Fig. 2B). Moreover, we found that CCNP downregulation decreased both OCT4 (*p*-values: A549 = 0.0195; LoVo = 0.0031; MCF7 = 0.0195) and SOX2 (*p*-values: A549 = 0.0112; LoVo = 0.0075; MCF7 = 0.0042) expression in the three cells lines (Fig. 2C).

We anticipated that genes regulated by CCNP are co-expressed with endogenous CCNP. Thus, to investigate whether these results are representative of CCNP in cancer in general, we extracted data from the Depmap portal (depmap.org/portal), which contains gene expression profiles of 1304 cell lines of different histological types (Expression Public 20Q2) [32].When cancer cells with the lowest and highest quarters of CCNP expression were grouped, the subset with a higher CCNP expression exhibited a significantly higher expression of OCT4 and SOX2 (*p* < 0.0001) (Fig. 2D), indicating that these correlations can be observed in several cancer models.

Given that our results suggest that CCNP regulates stemness in a pathological context, we next explored whether CCNP is involved in stemness regulation in a physiological context as well. For this purpose, we used CRISPR-Cas9 to generate induced-pluripotent stem cell (IPSC) lines harboring CCNP deletions, as confirmed by sequencing. In line with our previous results, we found that CRISPR-Cas9-mediated CCNP knockout (KO) was associated with a lower expression of OCT4 in the two IPSC clones analyzed (Fig. 2E), indicating that the ability of CCNP to regulate stemness is also likely to be relevant in contexts other than cancer.

### CCNP enhances resistance to chemotherapy

**Fig. 3:**
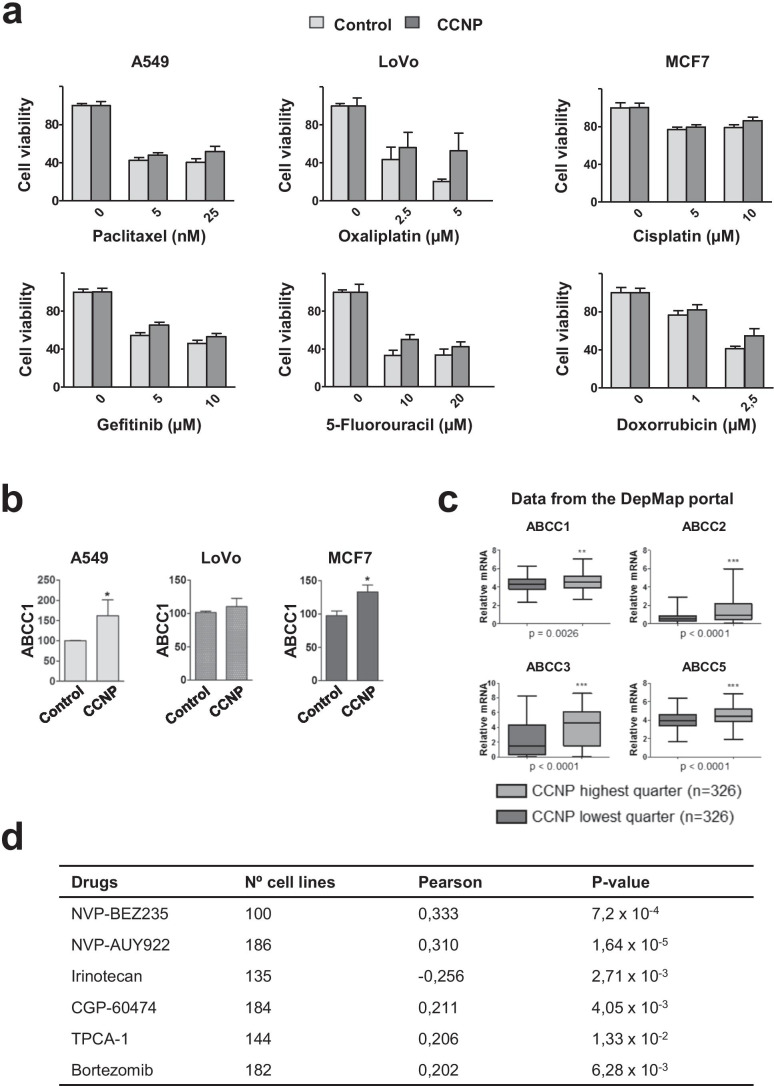
Overexpression of CCNP decreases cancer cell sensitivity to drug treatment. **a** A549, LoVo and MCF7 cells were transduced with an empty vector (control) or a CCNP expression construct and treated with the indicated concentrations of drugs. After 72 h, cell viability was measured by MTT assay. The columns represent the mean ± SEM of six independent experiments performed in triplicate. *p < 0.05, **p < 0.01 versus control, Mann–Whitney test. **b** RT-qPCR analysis of ABCC1 levels in A549, LoVo and MCF7 cells transduced with an empty vector (control) or a CCN expression construct. The columns represent the mean ± SEM of six independent experiments performed in triplicate. *p < 0.05 versus control, Mann–Whitney test. The results are expressed as percentage of the control. **c** Data from the Depmap portal were used to assess CCNP and ABC transporter correlations (n = 326 for each quarter). Data accessed on October 30th, 2020. **p < 0.01, ***p < 0.001, Mann–Whitney test. **d** Data regarding drug sensitivity were extracted from the Depmap portal (Sanger GDSC1 and GDSC2 IC50). The table shows the results for drugs that have been tested in 100 or more cell lines and display a Pearson Correlation > 0.2 or <  − 0.2

An important characteristic of CSCs is their ability to resist chemotherapy and to enable tumor regeneration when treatment stops [33]. To explore whether CCNP may induce resistance to chemotherapeutics, cells were transduced with an empty vector (control) or a CCNP expression construct and treated with different drugs for 72 h (Fig. 3A). We found that CCNP overexpression in A549 and LoVo cells conferred resistance to all the drugs tested (Fig. 3A and Supplementary Fig. S5A). Likewise, MCF7 cells were less sensitive to either cisplatin or doxorubicin treatment (Fig. 3A and Supplementary Fig. S5A). As these findings suggest that CCNP is associated with multidrug resistance, we monitored the effect of CCNP on the expression of members of the ATP-binding cassette (ABC) family of drug efflux transporters that have been implicated in resistance to these drugs [34]. CCNP upregulation increased the expression of several the drug efflux transporters tested (ABCC1 *p*-values: A549 = 0.0181; LoVo = 0.9358; MCF7 = 0.0247) (Fig. 3B and Supplementary Fig. S5B), supporting the notion that CCNP may contribute to the drug resistance of cancer cells.

To assess whether these observations can be reproduced in other cellular models, we extracted data from 1304 cancer cell lines (Depmap portal; Expression Public 20Q2) and confirmed that CCNP and ABC transporters are co-expressed (Fig. 3C). We also extracted data from the Depmap portal regarding drug sensitivity (Sanger GDSC1 and GDSC2 IC50) [35]. The data were filtered to consider only those drugs that have been tested in 100 or more cell lines and display a Pearson Correlation > 0.2 or < -0.2. We found that in 5 out of 6 drugs meeting these criteria, CCNP expression positively correlated with an increased half-maximal inhibitory concentration (IC50) (Fig. 3D). This observation supports the notion that CCNP upregulation may contribute to chemoresistance.

### CCNP regulates cancer cell stemness through activation of the WNT pathway

CCNP is one of the most poorly characterized members of the atypical cyclin family. Considering that the WNT pathway has amply been implicated in cancer stemness [36–40], we analyzed the effects of CCNP on WNT pathway components (http://www.gsea-msigdb.org/gsea/msigdb/genesets.jsp?collection=CP:BIOCARTA) in LoVo cells by RNA-sequencing. To provide robust results, we conducted two distinct genetic approaches, i.e., CCNP overexpression and downregulation. By doing so, we found a correlation between CCNP expression and the expression of GPC3 (False discovery rate, FDR = 0.00849), FRZB (FDR = 0.048), DKK4 (FDR = 0.0201) and CTHRC1 (FDR = 0.447), which are all related to cancer growth, stemness and invasiveness, in among others hepatocellular, ovarian and colorectal cancers [41–48]. Likewise, some proteins of the WNT pathway, which are known to function as cancer suppressors, were found to be overexpressed after CCNP knockdown. This was the case for SEMA5A (FDR = 0.0855), DRD2 (FDR = 0.00623) and SOSTDC1 (FDR = 0.0234), which have all been found to act as tumor suppressors in pancreas cancer, glioma, non-small cell lung cancer and thyroid cancer [49–54].

**Fig. 4:**
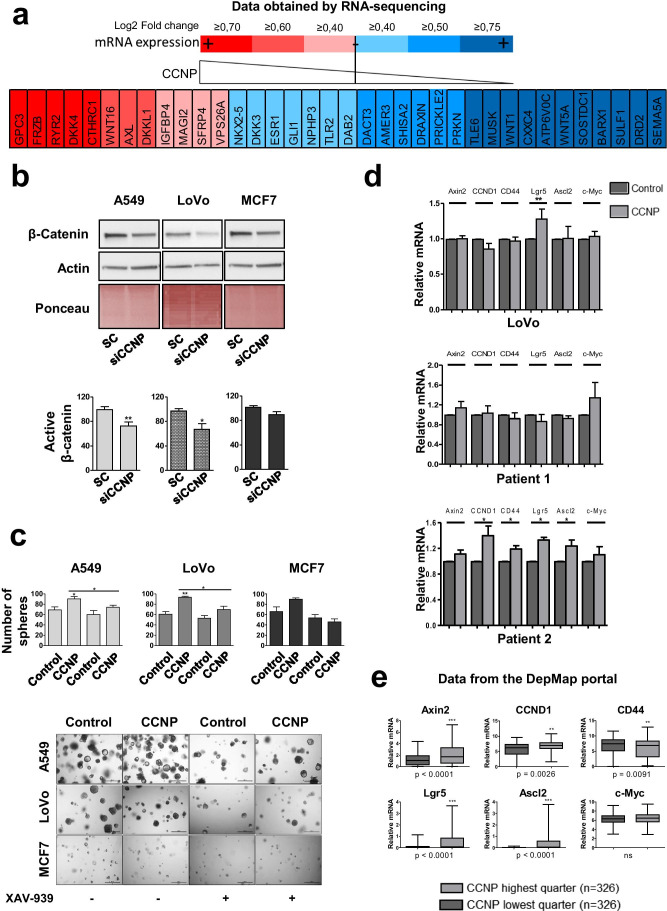
CCNP regulates oncosphere formation via the WNT signaling pathway **a** RNA-seq was performed to monitor the ability of CCNP to regulate WNT pathway components. **b** Cells were transfected with scrambled control (Sc) or siRNA targeting CCNP, after which the levels of activated β-catenin were monitored by Western blotting. The columns represent the mean ± SEM of nine independent experiments performed in triplicate. **c** Cells transduced with an empty (control) or a CCNP expression construct were seeded in Matrigel, treated with the WNT inhibitor XAV-939 and counted ten days later. The columns represent the mean ± SEM of ten independent experiments. Representative images of the experiments are shown below. **d** RT-qPCR of LoVo cells and patient-derived organoids transduced with empty (control) or CCNP expression vectors. *p < 0.05, **p < 0.01 versus control, Mann–Whitney test. **e** Data from the Depmap portal were used to assess correlations between CCNP and WNT-regulated gene expression profiles in human cell lines. Data accessed on June 6th, 2020. **p < 0.01, ***p < 0.001, Mann–Whitney test; *ns* not significant

In line with these results, we found that CCNP downregulation decreased the levels of activated β-catenin in LoVo (*p* = 0.0333) and A549 (*p* = 0.0043) cells (Fig. 4B). Conversely, we found that CCNP overexpression increased the level of activated β-catenin in A549 cells (Supplementary Fig. S6). More importantly, we found that the WNT pathway inhibitor XAV-939 blunted the increase in sphere number promoted by CCNP in the three cell lines tested (*p*-values vs. CCNP without XAV-939: A549 = 0.0235; LoVo = 0.0115; MCF7 = 0.0571) (Fig. 4C), supporting the notion that CCNP regulates cancer cell stemness through WNT pathway activation.

Next, we analyzed established stem cell markers and WNT pathway targets in LoVo cells and patient-derived organoids. In LoVo cells CCNP upregulation increased Lgr5 expression (*p* = 0.0097) and, while in Patient 1-derived organoids no significant effect on gene expression was found, in Patient 2 we found an increase in the mRNA levels of CCND1 (*p* = 0.0436), CD44 (*p* = 0.0436), Lgr5 (*p* = 0.0436) and Ascl2 (*p* = 0.0265) (Fig. 4D). Moreover, CCNP downregulation in Patient 2-derived organoids led to a significant decrease in Ascl2 mRNA expression (Supplementary Fig. S7). Finally, we monitored correlations between CCNP expression and WNT pathway targets in a large panel of cell lines using data from the DepMap portal. We found that high CCNP expression correlated with a high expression of Axin2 (*p* < 0.0001), cyclin D1 (CCND1) (*p* = 0.0026), Lgr5 (*p* < 0.0001) and Ascl2 (*p* < 0.0001), suggesting that CCNP and stemness are broadly intertwined in human cancer.

## Discussion

CCNP is an atypical cyclin that is clearly involved in cancer development [18]. It has previously been shown that CCNP upregulation is associated with lung cancer metastasis [24] and regional lymph node involvement in colorectal cancer patients [22]. Moreover, high levels of salivary CCNP mRNA have been found to markedly correlate with a worse prognosis in lung cancer patients [55]. Until now, however, a mechanistic explanation for the association between high CCNP expression levels and more aggressive cancer phenotypes has been missing. Here, we show that CCNP regulates cancer stemness through activation of the WNT pathway.

It is amply recognized now that cancer stem cells represent cells within the tumor mass that retain clonogenic potential and play pivotal roles in tumorigenesis, relapse and chemoresistance [3]. While multiple signaling pathways have been associated with cancer stemness, the underlying regulatory mechanisms are not yet completely understood, compromising the efforts to develop agents that specifically target cancer stem cells. Here we present CCNP as a novel WNT pathway activator that is specifically expressed in cancer cells [22], providing new opportunities for the design of therapeutic strategies.

Although more studies are warranted to provide further insight on the ability of CCNP to activate WNT, its action appears to be abolished by XAV-939, a tankyrase inhibitor that stimulates β-catenin degradation through the stabilization of axin [56], suggesting that CCNP may be acting upstream of it. Cyclin Y (CCNY), another member of the atypical cyclin subfamily, has been shown to promote phosphorylation of LRP6 in combination with CDK14 [57, 58] to enhance WNT signaling activity in dividing mammary stem/progenitor cells during mitosis [21]. Whereas the stemness-promoting actions of CCNY seem to be mediated by its interaction with a cyclin-dependent kinase (CDK), the CDK partner of CCNP (if any) is yet to be identified [17]. Recently, we performed a yeast 2-hybrid screen to identify atypical cyclin interactors, but no CCNP interactor was found using this strategy (Quandt et al., unpublished data). Thus, it remains unclear whether CCNP contributes to pluripotency through interaction with a CDK or through a mechanism other than phosphorylation of stem cell-specific cellular proteins. Given the correlation between high CCNP levels and CCND1 expression, it also remains to be explored whether proliferative actions of CCNP are related to WNT pathway activation, (Fig. 4D, E).

Although several signaling pathways have been implicated in stemness regulation, the WNT pathway has gained special interest due to its significance in several tissues [39]. A WNT signaling activator will likely be able to promote stemness in a large number of cancer types. However, considering that this is the first report of a role of CCNP in cancer stemness, it is difficult to speculate whether it may occur in other cancer types as well. Nevertheless, the analysis of a large panel of cell lines supports the notion that a correlation between CCNP and WNT target expression may occur in broad cancer cell contexts (Fig. 4E). Although CCNP induced the same phenotype in two patient-derived organoids, the underlying mechanisms may differ, as suggested by the differential effects of CCNP upregulation on the expression of WNT-related genes in the two patient samples analyzed (Fig. 4D). Our analysis at this point is limited by the small number of samples analyzed, and further studies are required to understand whether patient subpopulations display different expression signatures leading to the same phenotype. Our analysis was restricted to colon cancer patients considering that the role of stem cells in the origin of colon cancer is well established [59]. Studies in lung and breast cancer-derived samples are also warranted to confirm our findings in other tumor types.

Whereas CCNP overexpression was observed in highly prevalent cancers (Supplementary Fig. S1A), it was poorly expressed in normal tissues, except brain tissue [22, 24], suggesting that it may represent an ideal target for the design of novel therapies. As our results suggest that CCNP is associated with chemoresistance through the upregulation of ABC transporters (Fig. 3), CCNP targeting is likely to be beneficial in combination with chemotherapeutic agents to minimize resistance development. Considering that the self-renewal capacity of disseminated cancer cells is key to metastasis development [60], the unique features of CCNP may bring significant advances to the management of particularly aggressive cancers.

## Supplementary Information

Below is the link to the electronic supplementary material.

Supplementary file1 (PDF 802 kb)

## Data Availability

The analyzed data sets generated during the study are available from the corresponding authors upon reasonable request.
