# Supplementary material for: Atypical cyclin P regulates cancer cell stemness through activation of the WNT pathway
Source: Cell Oncol (Dordr). 2021 Oct 4;44(6):1273–86. doi: 10.1007/s13402-021-00636-7 (PMC8648692; doi:10.1007/s13402-021-00636-7)
Supplement: Supplementary file 1 — Supplementary file1 (PDF 802 kb) [file 13402_2021_636_MOESM1_ESM.pdf]

The atypical cyclin P regulates cancer cell stemness through the activation of the WNT pathway

Cellular Oncology

Abril Sánchez-Botet[1], Eva Quandt[1], Núria Masip[1], Rubén Escribá[2,3], Laura Novellasdemunt[4], Laura Gasa[1], Vivian S.W. Li[3], Ángel Raya[2,3,5], Josep Clotet[1], Mariana P.C. Ribeiro[1]

[1]Faculty of Medicine and Health Sciences, International University of Catalonia, 08195 Sant Cugat del Vallès, Barcelona, Spain.
[2]Regenerative Medicine Program, Bellvitge Institute for Biomedical Research (IDIBELL) and Program for Clinical Translation of Regenerative Medicine in Catalonia (P-CMRC), L'Hospitalet del Llobregat, Barcelona, Spain.
[3]Centre for Networked Biomedical Research on Bioengineering, Biomaterials and Nanomedicine (CIBER-BBN), Madrid, Spain.
[4]Stem Cell and Cancer Biology Laboratory, The Francis Crick Institute, London, UK.
[5]Catalan Institution for Research and Advanced Studies (ICREA), Barcelona, Spain.

Correspondence to: Josep Clotet Erra and Mariana Ponte Cardoso Ribeiro , Universitat Internacional de Catalunya, jclotet@uic.es and mpontecardosoribeiro@uic.es.

a

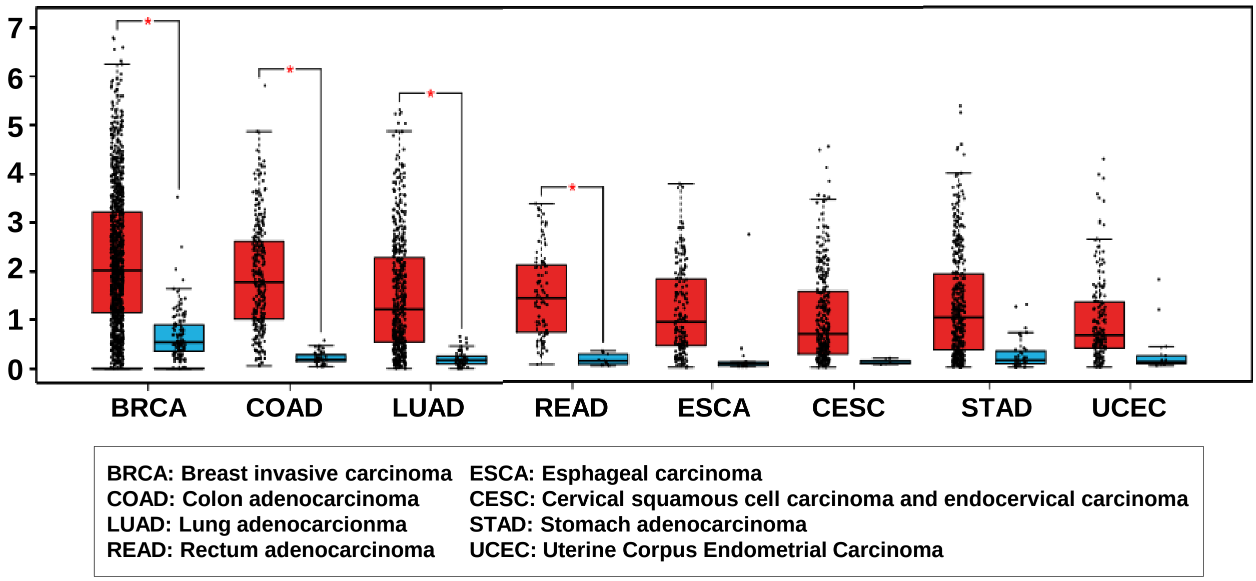

b

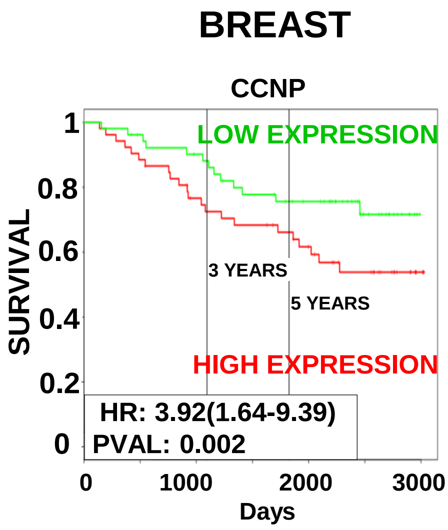

Supplementary Fig. S1 CCNP is upregulated in tumor tissues. (A) mRNA expression in tumor (red boxes) and adjacent healthy (blues boxes) tissues in various types of cancers according to the GEPIA database (http://gepia.cancer-pku.cn/). Data accessed in August 2018. (B) Survival curves in breast cancer patients presenting high and low levels of CCNP expression according to the PROGgeneV2 tool (GSE42568 database, n = 104). Data accessed in July 2018.

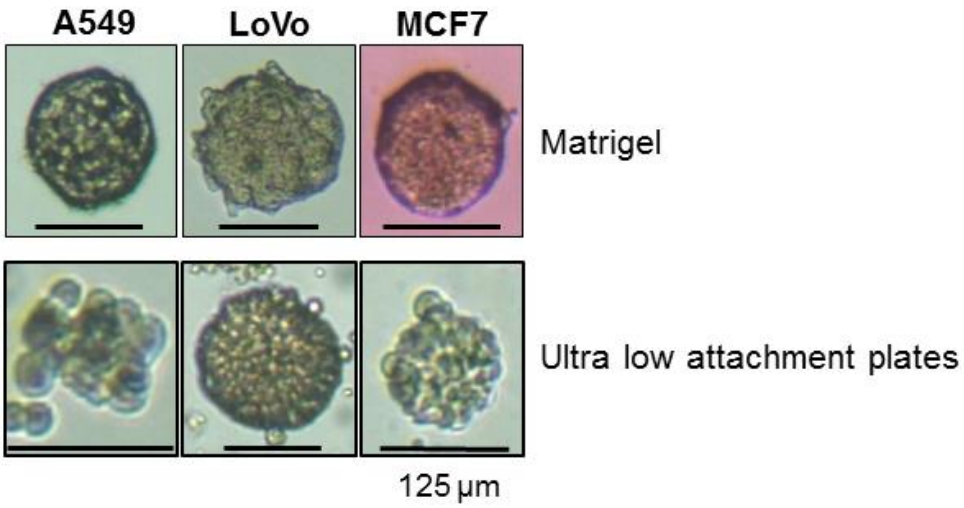

Supplementary Fig. S2 Comparison of spheroid morphology. The image shows representative images of spheroids obtained by seeding in matrigel or ultra-low attachment plates.

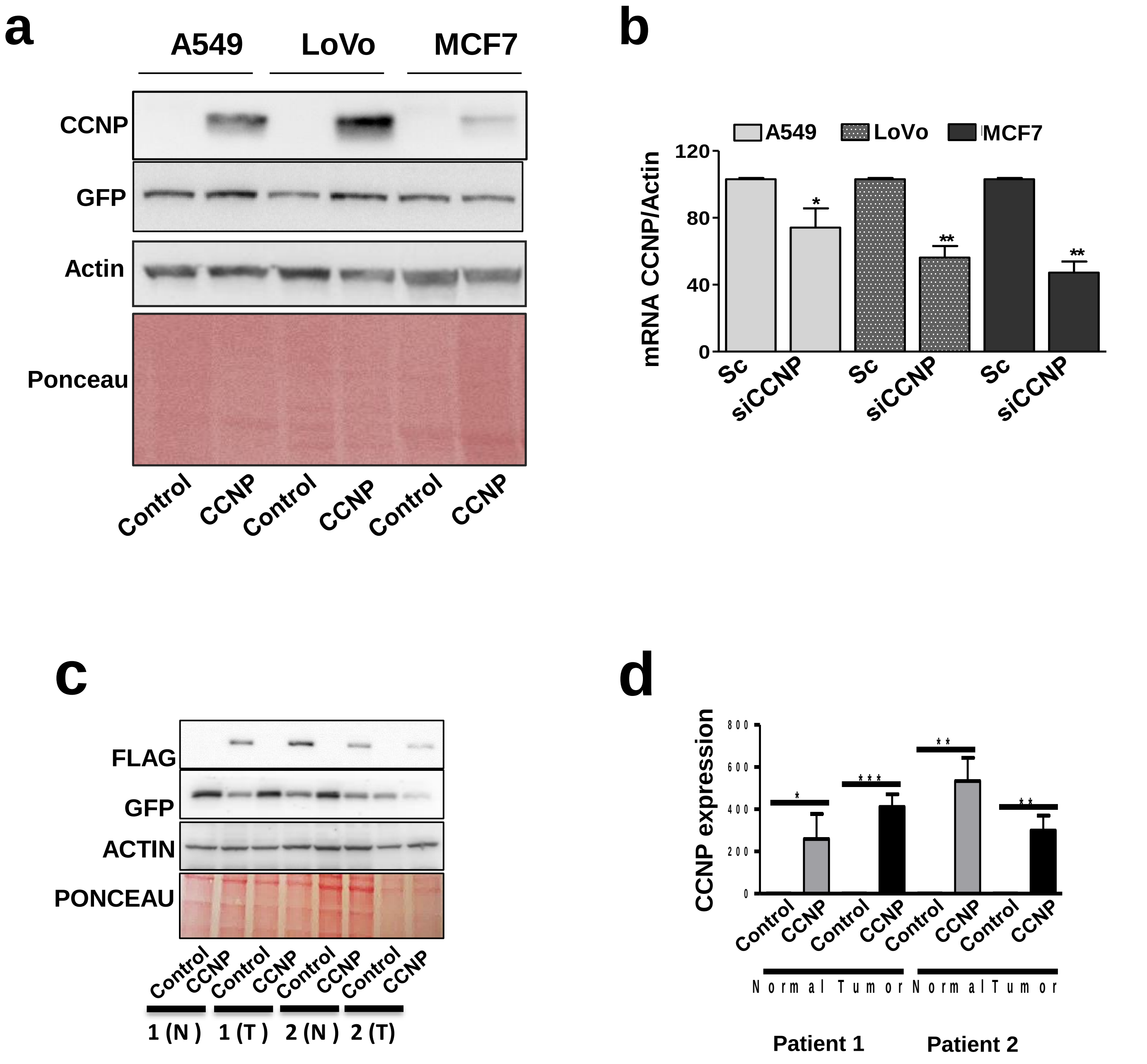

Supplementary Fig. S3 CCNP overexpression and downregulation in cancer cell lines and patient-derived organoids. (A) Cells were transduced with an empty lentiviral vector (control) or with CCNP expression constructs. Protein overexpression was confirmed by Western blot analysis. (B) Cells were transfected with scrambled control (Sc) or siRNA targeting siRNA (siCCNP) at 50 nM. After 72 h, CCNP expression was monitored by RT-qPCR. The columns represent the mean ± SEM of five independent, ** P <0.01, * P <0.05 vs. scrambled control (Sc), Mann-Whitney test. (C-D) Normal and tumor-derived organoids of patients 1 and 2 were transduced empty vector (control) or CCNP-expression construct. CCNP-Flag expression was followed by western blot (C) and RT-qPCR (D)

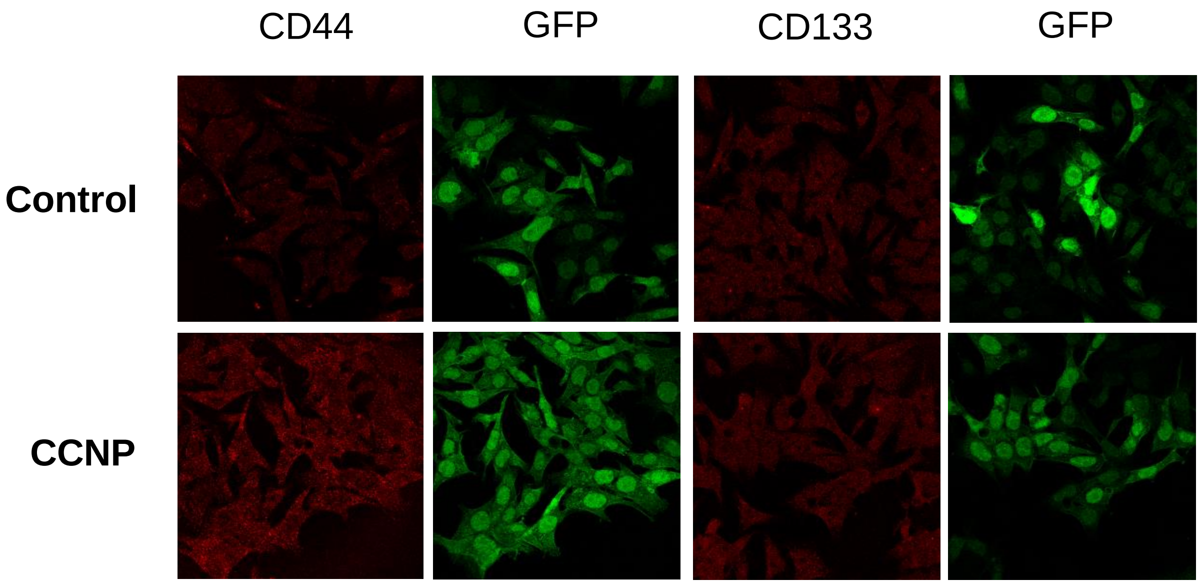

Supplementary Fig. S4 Immunofluorescence of stemness markers CD44 and CD133 in LoVo cells overexpressing CCNP. Cells were transduced with an empty vector or CCNP expression construct and seeded on poly-lysine coated coverslips. Forty-eight hours later, cells were fixed in cold paraformaldehyde, blocked for 1 h, and incubated for 1 h with CD44 or CD133 antibodies. Coverslips were then washed several times with PBS and incubated with anti-rabbit fluorescent secondary antibody.

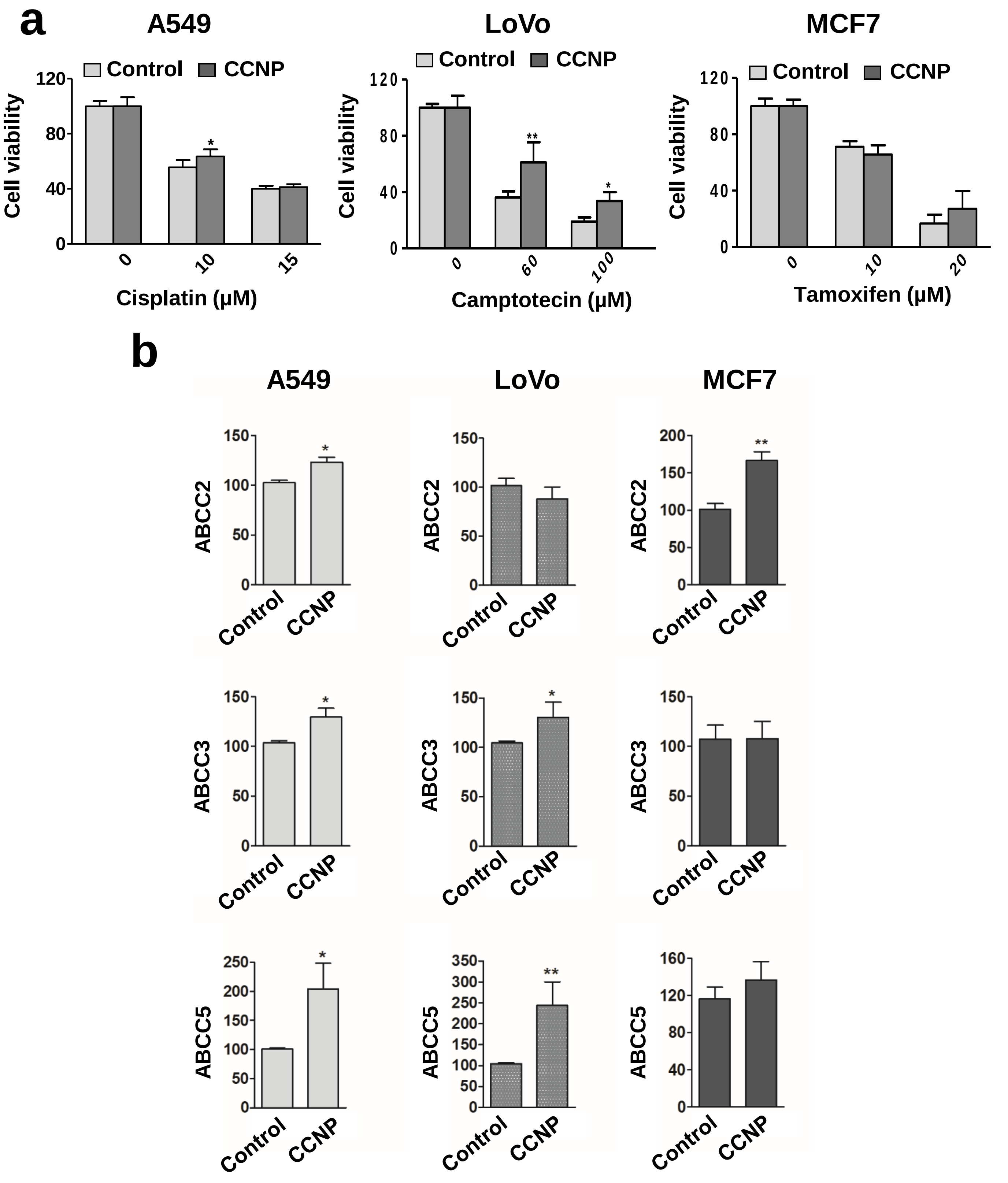

Supplementary Fig. S5 Overexpression of CCNP decreases cancer cell sensitivity to drug treatment. Cells were transduced with an empty vector (control) or with a CCNP-expressing construct and treated with the indicated concentrations of drugs. Cell viability was measured by the MTT assay (A), and the expression of ABC transporters was monitored by RT-qPCR (B). The columns represent the mean ± SEM of six independent experiments performed in triplicate. *P <0.05, **P <0.01 vs control. The results are represented as a percentage of control

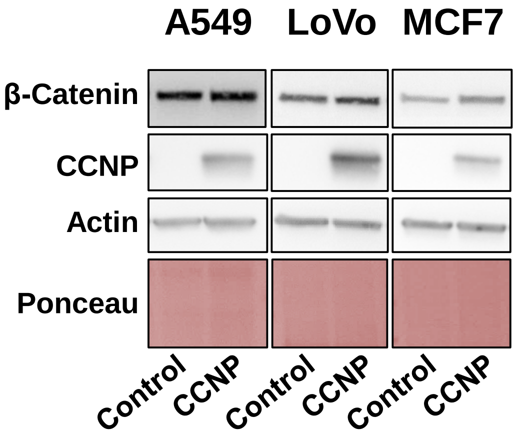

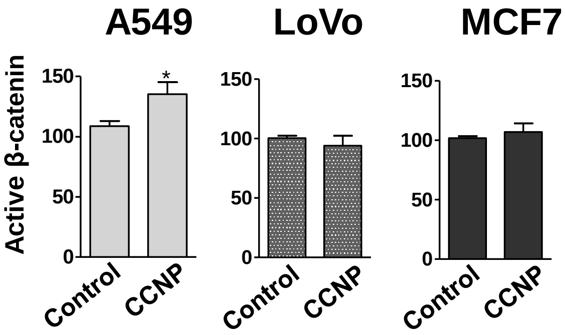

Supplementary Fig. S6 Overexpression of CCNP decreases cancer cell sensitivity to drug treatment. Cells were transduced with an empty vector (control) or with a CCNP-expressing construct, and the levels of active β-catenin were monitored by western blot. Representative images are shown in the left panel, whereas the right panel shows activated β-catenin quantification. The columns represent the mean ± SEM of nine independent experiments performed in triplicate. *P <0.05 vs. control, Mann-Whitney test. The results are represented as a percentage of control.

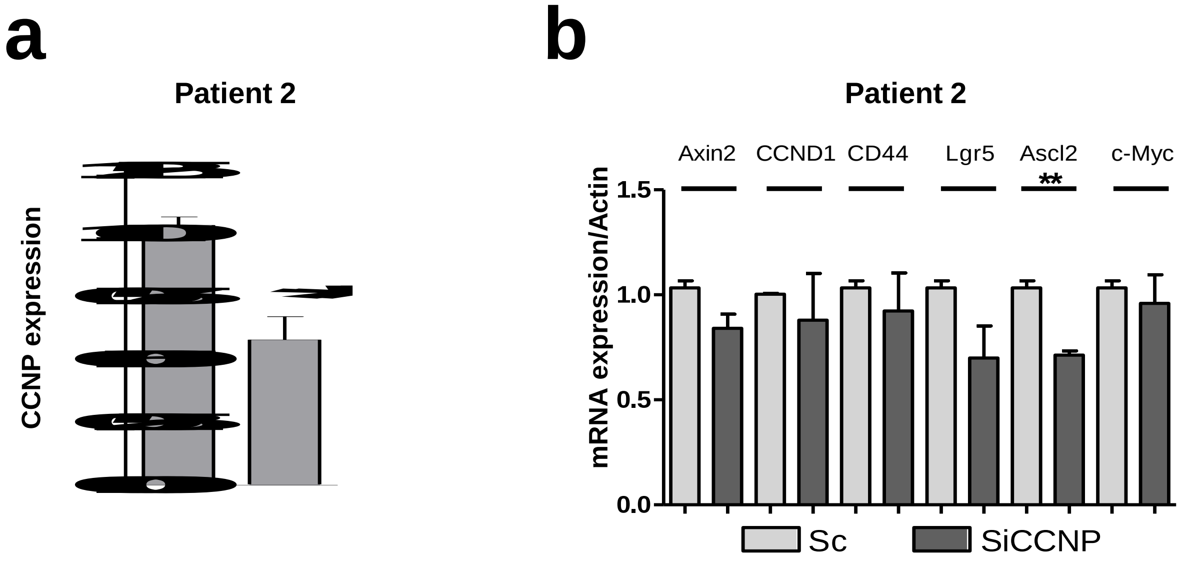

Supplementary Fig. S7 Knockdown of CCNP reduces the expression of Ascl2 in Patient 2 tumor-derived organoids. Tumor organoids of patient 2 were transfected with scrambled control (Sc) or with siRNA targeting CCNP (siCCNP). Knockdown efficiency (A) and the expression of stem cell and WNT targets (B) were evaluated by RT-qPCR. The columns represent the mean ± SEM of three independent experiments, ** P <0.01 vs. control, T-student test. The results are represented as a percentage of control

Cell line	Source	Catalog nº	STR
A549	European Collection of Authenticated Cell Cultures	86012804	**Amelogenin:** X,Y; **CSF1PO:** 10,12; **D13S317:** 11; **D16S539:** 11,12; **D5S818:** 11; **D7S820:** 8,11; THO1: 8,9.3; **TPOX:** 8,11; **vWA:** 14
HEK293-T	Authenticated by the American Type Culture Collection		**CSF1PO:** 11,12; **D13S317**: 12,14; **D16S539**: 9,13; **D5S818**: 8,9; **D7S820**: 11; **TH01**: 7, 9.3; **TPOX**: 11; **vWA**: 16,19; **Amelogenin**: X
HT-29	European Collection of Authenticated Cell Cultures	91072201	**Amelogenin:** X; **CSF1PO:** 11,12; **D13S317:** 11,12; **D16S539:** 11,12; **D5S818:** 11,12; **D7S820:** 10; **THO1:** 6,9; **TPOX:** 8,9; **vWA:** 17,19
LoVo	Authenticated by the American Type Culture Collection		**Amelogenin:** XY; **CSF1PO:** 11,13,14; **D13S317:** 8, 11; **D16S539:** 9, 12; **D5S818:** 11, 13; **D7S820:** 9.3,10, 11; **THO1:** 9.3; **TPOX:** 8,9; **vWA:** 17,18
MCF7	Authenticated by the American Type Culture Collection		**Amelogenin:** X; **CSF1PO:** 10; **D13S317:** 11; **D16S539:** 11,12; **D5S818:** 11,12; **D7S820:** 8,9; **THO1:** 6; **TPOX:** 9,12; **vWA:** 14,15
MDA-MB-231	Authenticated by the American Type Culture Collection		**Amelogenin:** X; **CSF1PO:** 12,13; **D13S317**: 13; **D16S539:** 12; **D5S818:** 12; **D7S820:** 8,9; **THO1:** 7,9.3; **TPOX:** 8,9; **vWA:** 15,18
NCI-H1395	American Type Culture Collection	CRL-5868	**Amelogenin:** X; **CSF1PO:** 12; **D13S317:** 10,14; **D16S539:** 11,13;**D5S818:** 12; **D7S820:** 8; **THO1:** 6,9.3; **TPOX:** 8; **vWA:** 14,17

Supplementary Table S1 List of cell lines used.

Gene	Sequence
18S Forward	CTACCACATCCAAGGAAGGCA
18S Reverse	TTTTTCGTCACTACCTCCCCG
CCNP Forward	CTGGTGGTAGACTGGCTGGT
CCNP Reverse	AGCACGCACTCTTCCATTTT
OCT4 Forward	GTGGAGAGCAACTCCGATG
OCT4 Reverse	TGCAGAGCTTTGATGTCCTG
Sox2 Forward	TGG CGA ACC ATC TCT GTG GT
Sox2 Reverse	CCA ACG GTG TCA ACC TGC AT
Nanog Forward	CAG AAG GCC TCA GCA CCT AC
Nanog Reverse	ATT GTT CCA GGT CTG GTT GC
ABCC1 Forward	TGCCCTACCTGACCCTCGGC
ABCC1 Reverse	ACACCCAGTCAGGCTCCGCA
ABCC2 Forward	TCTCTCGATACTCTGTGGCAC
ABCC2 Reverse	CTGGAATCCGTAGGAGATGAAGA
ABCC3 Forward	GCTGGGCTGGGAAACCGGAC
ABCC3 Reverse	TGGGTGCAGCCTGGAACAGC
ABCC5 Forward	CCCGCCCCAGGCAGGGAATG
ABCC5 Reverse	TGTGTCCTGACGGCGTCTCCTT

Supplementary Table S2 List of RT-qPCR primers

Cat. No	Antibody	Species	Company
2956	GFP	Rabbit	Cell Signaling
HPA045615	CCNP	Rabbit	Sigma
GTX627419	OCT-4	Mouse	GeneTex
Sc-7297	CD44	Mouse	Santa Cruz
E-AB-33462	CD133	Rabbit	Elabscience
Sc-374429	INTEGRIN β	Mouse	Santa Cruz
8814	ACTIVATED β -CATENIN	Rabbit	Cell Signaling
Sc-58628	Na+ K+ ATPase	Mouse	Santa Cruz
CSB-PA00025A0Rb	GAPDH	Rabbit	CusAb
Sc-374015	LAMIN B	Mouse	Santa Cruz
Sc-17824	SP1	Mouse	Santa Cruz
F3165	FLAG	Mouse	Sigma-Aldrich
A5316	β-ACTIN	Mouse	Sigma-Aldrich

Supplementary Table S3 List of antibodies used
